# Manufacture of Bilayered Composite Hydrogels with Strong, Elastic, and Tough Properties for Osteochondral Repair Applications

**DOI:** 10.3390/biomimetics8020203

**Published:** 2023-05-16

**Authors:** Hui Yao, Congcong Wang, Yuchen Zhang, Ying Wan, Qing Min

**Affiliations:** 1 School of Pharmacy, Hubei University of Science and Technology, Xianning 437100, China; 2 Hubei Engineering Research Center of Traditional Chinese Medicine of South Hubei Province, Xianning 437100, China; 3 College of Life Science and Technology, Huazhong University of Science and Technology, Wuhan 430074, China

**Keywords:** chitosan, silk fibroin, hyaluronic acid, bioglass nanoparticles, layered composite hydrogel, mechanical property

## Abstract

Layered composite hydrogels have been considered attractive materials for use in osteochondral repair and regeneration. These hydrogel materials should be mechanically strong, elastic, and tough besides fulfilling some basic requirements such as biocompatibility and biodegradability. A novel type of bilayered composite hydrogel with multi-network structures and well-defined injectability was thus developed for osteochondral tissue engineering using chitosan (CH), hyaluronic acid (HA), silk fibroin (SF), CH nanoparticles (NPs), and amino-functionalized mesoporous bioglass (ABG) NPs. CH was combined with HA and CH NPs to build the chondral phase of the bilayered hydrogel, and CH, SF, and ABG NPs were used together to construct the subchondral phase of the bilayer hydrogel. Rheological measurements showed that the optimally achieved gels assigned to the chondral and subchondral layers had their elastic moduli of around 6.5 and 9.9 kPa, respectively, with elastic modulus/viscous modulus ratios higher than 36, indicating that they behaved like strong gels. Compressive measurements further demonstrated that the bilayered hydrogel with an optimally formulated composition had strong, elastic, and tough characteristics. Cell culture revealed that the bilayered hydrogel had the capacity to support the in-growth of chondrocytes in the chondral phase and osteoblasts in the subchondral phase. Results suggest that the bilayered composite hydrogel can act as an injective biomaterial for osteochondral repair applications.

## 1. Introduction

Articular cartilage is a tissue with avascular features, and it has the limited ability to regenerate once damaged, especially for cartilage damage with a large defect size [1]. The cartilage damages often induce osteochondral defects (OCDs), and in addition, some subchondral bone-associated diseases, such as osteochondritis dissecans, osteonecrosis, and osteochondral fracture, can also cause OCDs [1,2]. Several kinds of surgical interventions, majorly including arthroscopic debridement, microfracture, and allograft or autograft implantation, are commonly used for OCD treatment [1,2]. Nevertheless, the treatments associated with these techniques remain frequently unsatisfying, and many clinical outcomes are considered unsuccessful based on long-term convalescence evaluation [2,3]. It is thus imperative to develop alternative treatments.

At present, a tissue engineering technology that usually employs selected cells, biomaterials, and bioactive molecules has emerged as a promising approach for repairing OCDs [3]. The osteochondral unit in diarthrodial joints is a multiphasic tissue that is consisted of two main tissue types: the hyaline cartilage and the subchondral bone. It is known that support from healthy subchondral bone is one of the essential requirements for articular cartilage repair, and the functional recovery of joints ultimately relies on the rehabilitation of both tissues [3,4]. Accordingly, various types of gradient or layered scaffolds have been developed for the treatment of OCDs by repairing both cartilage and bone tissues simultaneously [4,5]. Besides the solid layered scaffolds, injectable polymer hydrogels with biocompatible and biodegradable features have also aroused a lot of interest in the repair of OCDs, because they can form into self-supporting objects with the devised composition and architecture in a minimally invasive manner for filling complex tissue cavities [6]. Besides these, hydrogels can readily function as carriers for the delivery of cells, drugs and active agents due to their interconnected porous structures and high water retention, which is conducive to in situ construction of a dynamic microenvironment for mimicking the extracellular matrix (ECM) of osteochondral tissues [6,7].

To date, various kinds of natural polymers, majorly including collagen, gelatin, silk fibroin (SF), chitosan (CH), dextran, alginate, and hyaluronic acid (HA), have been widely explored for hydrogel applications, because they usually show good biocompatibility, easy biodegradation, and better biological performance than many synthetic polymers [8]. HA is an essential component of the ECM with multifaceted functions in mediating cellular migration, matrix development and organization. Hence, HA-based hydrogels have now found a variety of applications in tissue engineering [9]. CH has a chemical structure similar to glycosaminoglycans (GAG) that widely exist in ECM of different human tissues and is thus favorable for tissue repair and regeneration in the form of hydrogel [10]. Despite the wide usability of CH or HA hydrogels, the CH hydrogel often shows fragility and the HA hydrogel usually has low mechanical strength with a high swelling ratio and fast in vivo degradation.

SF is a natural fibrous protein with several demonstrated advantages such as low inflammation response, promotion of cell adhesion, supporting cell growth, and facilitation of three-dimensional colonization for many types of cells [11]. SF can be processed into hydrogels with tunable mechanical properties ranging from soft to stiff or from weak to highly strong, with dependence on the crosslinking method and amount of crosslinker applied [12,13]. Among available crosslinkers for SF hydrogels, horse radish peroxidase (HRP) and hydrogen peroxide (H_2_O_2_) have been widely employed as crosslinking co-agents for catalyzing the crosslinking reaction between the tyrosine residues of SF chains, since the involved crosslinking reaction can be conducted on the physiological conditions with adequate safety by controlling the amount of HRP and H_2_O_2_ [13].

Concerning the mechanical properties only, articular cartilage faces a rather complex mechanical environment in which articular cartilage has to repeatedly experience various forces, such as tension, shear, and pressure [14,15]. Accordingly, the hydrogel that is aimed to use for repairing osteochondral defects should be mechanically strong, elastic, and tough [15]. Many studies have suggested that polymer hydrogels with dual- or multi-networks can be remarkably enhanced in mechanical properties and degradation tolerance through various types of interactions between molecular chains and crosslinkable groups, compared to the hydrogels with a single network [16,17,18]. Taking account of the merits of CH, HA, and SF, it would be feasible to build strong, elastic, and tough multi-network hydrogels with biphasic structures by optimally combining them while employing suitable crosslinkers for building multiple networks inside the gels.

With the above-mentioned information in mind, an attempt was made in this study to build a novel type of bilayered composite hydrogel with multi-network structure and injectability for osteochondral tissue engineering using CH, SF, HA as well as CH nanoparticles (NPs) and amino-functionalized mesoporous bioglass (ABG) NPs. One layer was constructed with CH, HA, and CH NPs, and it was assigned to the chondral layer (top layer). Another layer was built with CH, SF, and ABG NPs, and it was designated as the subchondral layer (bottom layer). The CH NPs in the chondral layer play two roles: (i) they serve as a component for the multi-network construction, and (ii) they could function as a potential carrier for loading certain drugs or bioactive molecules to endue the chondral layer with more functionality. As regards the mesoporous ABG NPs in the subchondral layer, they also make several contributions to this layer. The surface amino groups of ABG NPs can be used for the crosslinking reaction, and thus, contribute to the formation of amino-involved networks. The pores of ABG NPs would be useful for loading certain agents, enriching the function of the layer. More importantly, certain dissolution products of ABG NPs, such as bioactive Si and Ca ions, have the ability to stimulate osteogenesis [19]. In the first step of this work, the preparation and characterization of bilayered composite hydrogels were mainly investigated. The optimally constructed bilayered hydrogels showed strong, elastic, and tough characteristics and also, had a capacity for supporting cell growth. The results suggest that the bilayered hydrogels have the potential for osteochondral repair applications.

## 2. Materials and Methods 

### 2.1. Materials

Chitosan (CH) and HRP (300 U/mg) were procured from Aladdin Inc. (Shanghai, China). After treatment with a NaOH aqueous solution (50 wt%) [20], the degree of deacetylation and viscosity-average molecular weight of resulting CH were measured to be about 95.4% and 3.8 × 10^4^, respectively. Glycerolphosphate (GP) and poly(ethylene glycol) diglycidyl ether (PEGDE, Mn of PEG: 2000), and sodium tripolyphosphate (TPP) were purchased from Sigma-Aldrich (Shanghai, China). HA (sodium salt, Mw: 70 kDa) was procured from Sangon Biotech Ltd. (Shanghai, China). Other chemicals were of analytical grade and were purchased from Sinopharm (Shanghai, China). 

SF was isolated from cocoons using the method previously described in our study [21]. The obtained dilute SF solution was concentrated with a 30% PEG20000 solution, and the resulting SF solution was stored at 4 °C for further use.

ABG NPs were prepared using a two-step method. Mesoporous bioglass (BG) NPs were first synthesized, and these BG NPs were then reacted with 3-(aminopropyl) triethoxysilane (APTES) to produce ABG NPs. Details for the synthesis of ABG NPs can be found in our previous study [22].

### 2.2. Preparation of CH NPs

CH NPs were prepared using a reported method with some modifications [23]. CH was dissolved in a 1% aqueous HCl solution to prepare a 1% CH solution. TPP was dissolved in distilled water at a concentration of 1.2%, and this TPP solution was further added with a small amount of PEGDE to reach a PEGDE concentration of 0.1%. To 20 mL of CH solution, the TPP solution was introduced at a rate of 20–30 drops per minute with stirring, and the addition of the TPP solution was stopped once the reaction system was visualized to be opalescent. The opalescent suspension was further stirred for 30 min, and the resulting mixture was moved to a membrane tube (MWCO 3.5K) and dialyzed against distilled water for 72 h. The obtained product was lyophilized to achieve CH NPs. 

### 2.3. Preparation of Hydrogels for Chondral Phase

CH, HA, and CH NPs were used to prepare composite solutions. In brief, 90 mg of CH was introduced into a vial that was filled with 3.1 mL of 0.1 M HCl in five runs (18 mg each) with stirring to dissolve CH. To a HA solution (2.5%, 1.2 mL) in distilled water, 50 mg of CH NPs was introduced in two runs (25 mg each) with stirring for 5 min in each run, and the mixture was then added to the vial containing the CH solution with stirring. Subsequently, the vial was moved to a water bath that was maintained at 4 °C, followed by the addition of an aqueous GP solution (56%, 0.5 mL) with stirring for 3 min. After that, 0.2 mL of aqueous PEGDE solution (2.5%) was added to the vial with stirring additionally for 3 min to achieve the composite solution. By varying the amount of CH NPs fed, different kinds of composite solutions were produced. These composite solutions were further processed into gels by placing them in a water bath at 37 °C. Gelation time was assessed with a tube-inverting method. During the incubation of composite solutions for gelling, the fluidity of the solutions was checked regularly by inverting the vial. The estimated gelation time was recorded from the start of the vial incubation until the solution stopped flowing. These composite solutions were also subjected to pH-value measurements using an FE20 pH meter with a viscotrode (Mettler Toledo Technology Ltd., Shanghai, China). Several kinds of composite gels with designed formulations were prepared, and the optimal one will be used for building the chondral phase of bilayered gel. Some gels without embedding CH NPs were also prepared following the same protocol and used as controls.

### 2.4. Preparation of Hydrogels for Subchondral Phase

CH, SF, and ABG NPs were used to prepare different composite solutions. A typical procedure was shown below. A total of 60 mg of CH was introduced into a vial that was filled with 4.2 mL of 0.1 M HCl in three runs (20 mg each) with stirring to dissolve CH. ABG NPs (50 mg) were then added to the vial in two runs (25 mg each) with sonication treatments for 5 min in each run. Subsequently, 40 mg of CH was added to the vial in two runs (20 mg each) with stirring to further dissolve CH. After that, 50 mg of SF was added to the vial with stirring to dissolve SF. The vial was placed in a water bath that was maintained at 4 °C, and then, 50 μL of H_2_O_2_ solution (100 mmol/L) and 50 μL of HRP solution (200 U/mL) were added with stirring for 2 min. Afterward, 0.5 mL of GP solution (56%) was added to the vial with stirring for 3 min, followed by the addition of 0.2 mL of PEGDE solution (2.5%) with stirring additionally for 3 min to achieve the needed composite solution. By changing the feed amount of ABG NPs while keeping the percentage of other components constant, different kinds of composite solutions were prepared. These composite solutions were further incubated at 37 °C for gelling. The gelation time and pH value of composite solutions were measured using the same methods mentioned above. Several kinds of gels were produced using the designed formulations. Among them, the gels without incorporating ABG NPs were used as controls, and the optimally achieved gel with the incorporation of ABG NPs was employed to construct the subchondral phase of bilayered gels.

### 2.5. Preparation of Bilayered Hydrogels

Bilayered hydrogels were built using homemade orifice molds in which each circular hole has a diameter of 10 mm and a height of 4 mm. The composite solution selected for use in the subchondral phase (bottom layer) of bilayered gel was injected into the holes of the mold to reach a height of 2 mm, and the mold was placed in an incubator and maintained at 37 °C for 2 min to partially thicken. Subsequently, the composite solution assigned to the chondral phase was injected atop the bottom layer, and the mold was further incubated at 37 °C for full gelling.

### 2.6. Measurements of NPs and Gels

The morphology of NPs was viewed using a transmission electron microscope (TEM, Tecnai, FEI, Hillsboro, OR, USA). The hydrodynamic size and zeta (ζ) potential of NPs were detected with a dynamic light scattering (DLS) instrument (Nano-ZS90, Malvern, Malvern, Worcestershire, UK). Regarding the isotherm detections of NPs, these NPs were first dried at 100 °C for 12 h under reduced pressure and degassed at 120 °C for 12 h before they were subjected to nitrogen adsorption–desorption measurements using a pore size analyzer (ASAP 2020 Plus, Micromeritics, Norcross, GA, USA). The BET method was used to determine the specific surface areas of NPs, whereas the BJH method was used to calculate the pore size and volume of NPs. Some dry gels were prepared by freezing the prepared gels in liquid nitrogen first and then lyophilizing the frozen samples. The dry gel samples were viewed using a scanning electron microscope (SEM, Quanta 200, FEI, Eindhoven, The Netherlands). Three hundred pores that were randomly selected from the SEM images of each sample were measured, and their size distribution and average pore size were determined with the virtue of analysis software. 

The porosity of dry gel samples was measured using a specific gravity bottle method based on the following formula:

Porosity (%) = [(W_2_ − W_3_ − W_S_)/(W_1_ − W_3_)] × 100 (1)

where W_1_ is the weight of the specific gravity bottle filled with ethanol; W_2_ is the weight of the specific gravity bottle including ethanol and dry gel (ethanol above the mark was removed); W_3_ is the weight of the specific gravity bottle taken out of the ethanol-saturated gel; W_S_ is the weight of dry gel.

Cylindrical gels (1 mL, 10 mm in diameter) were used to assess their swelling. Briefly, the gel samples were immersed in PBS (5 mL) at ambient temperature until their swelling equilibrium was reached. The weight of gels (W_s_) was measured after the removal of superficial water with filter paper, followed by freeze-drying for determining the weight of dry gels (W_d_). The swelling index (SI) of gels was calculated by the following formula:

SI = (W_s_ − W_d_)/W_d_ (2)

### 2.7. Rheological Tests

Rheological tests were carried out using a rheometer (Kinexus Pro KNX2100, Southborough, MA, USA) equipped with a parallel-plate sample holder. Frequency sweep tests for elastic modulus (G′) and viscous modulus (G″) of gels were conducted in a range between 0.1 and 100 Hz at 37 °C at a 1% constant strain. In the case of strain sweep, G′ and G″ of gels were detected by setting the temperature at 37 °C and frequency at 1 Hz, respectively. Shear viscosity tests were performed at 25 °C using liquid samples, and the shear rate was set in a range between 0.1 and 100 s^−1^. The oscillatory shear rheological measurements for gel samples were performed with strain sweeps from 0.01 to 10 % by setting the temperature at 37 °C and frequency at 1 Hz. The storage shear modulus (G_s_) of the gels at 1 % strain was used for data analyses [24,25]. For each kind of sample, the rheological measurement was performed in triplicate.

Based on SI and G_s_, the crosslinking density (*ρ*) of gel can be estimated with the following formula provided that the Poisson’s ratio of gel is set at 0.5 [26,27]:

*ρ* = G_s_/RTΦ ^1/3^ (3)

where R is the gas constant (8.314 J/ mol·K), T is the absolute temperature (298K), and Φ is the volume fraction of polymer in the swollen gel and determined by SI.

### 2.8. Mechanical Tests

Compression tests were carried out using a testing machine (Instron 5944, Norwood, MA, USA) in an unconfined manner. Cylindrical gel samples with a diameter of 10 mm and a height of 4 mm were prepared using homemade molds, and compression tests were conducted at a strain rate of 10%/min. The gel samples were compressed to 10 loading–unloading cycles employing a strain rate of 10%/min and a strain of 40% to determine their hysteresis curves. After each cyclic test, the gel samples were allowed to stay at zero position for 10 min for 3-dimensional restoration before the next text started. The compressive modulus (E) of gels was calculated from the slope of linear fitting of the stress–strain curves at 5% strain and blow.

Strain-recovery rate (R_s_%) is calculated as follows:

R_s_(%) = [h(t)/h_0_] × 100% (4)

where h_0_ is the original height of the gel sample, and h(t) is the recovered height of the gel sample after unloading.

### 2.9. Release of Ions

Cylindrical bilayered gel samples (diameter: 10 mm; height: 4 mm) were placed in a series of PBS (3 mL)-filled vials with shaking at 60 rpm and 37 °C. At prescribed time points, 1 mL of supernatant was withdrawn, and the vials were added with the same volume of fresh PBS. The concentration of ions in the collected supernatants was detected using inductively coupled plasma optical emission spectrometry (ICP-OES, Prodigy Plus, Leeman Labs, Hudson, NH, USA). Ion release of ABG NPs with the same amount of NPs in the subchondral phase (bottom layer) of bilayered gel was also measured for comparison. 

### 2.10. Cell Culture

Mouse articular chondrocytes and mouse osteoblasts were purchased from Newgainbio Tech Inc. (Wuxi, China). The cells were expanded using the DMEM medium with the supplement of 10% fetal bovine serum and 1% penicillin/streptomycin. Cell culture was conducted in a 5% CO_2_ humidified atmosphere at 37 °C. Cell suspensions in PBS were prepared for further use.

Cell-containing hydrogels were used for evaluating the viability and proliferation of the seeded cells. Chondrocytes were mixed with the sterilized composite solution matching with the chondral phase to produce several kinds of chondrocyte-containing mixtures. Osteoblast-containing mixtures were also prepared with the same method but choosing the sterilized composite solution corresponding to the subchondral phase. For the live/dead staining essay, the prepared cell-containing mixtures, 100 μL apiece (2 × 10^5^ cells for chondrocytes, 10^5^ cells for osteoblasts), were placed in confocal dishes and incubated at 37 °C for gel formation. The gels were then cultured in a complete medium for various periods up to 7 days with medium replacement every 2 days. At prescribed time points, the medium was aspirated, and the gels were cultured with serum-free medium containing calcein acetoxymethyl ester and propidium iodide in the dark for cell staining. The gels were washed PBS and imaged using a confocal microscope (LSM 510 META, Zeiss, Shanghai, China). 

The DNA content in cell-gel constructs was measured to assess cell proliferation. The osteoblast-containing mixture was first injected into the holes of homemade orifice mold (10 mm in diameter and 4 mm in height) to reach a height of 2 mm for functioning as the bottom layer (10^6^ cells), and the mold was placed in an incubator and maintained at 37 °C for 2 min to partially thicken. Subsequently, the chondrocyte-containing mixture (2 × 10^6^ cells) was injected atop the bottom layer, and the mold was further incubated at 37 °C for full gelling. The obtained cell–gel constructs were cultured with a complete culture medium using a homemade two-compartment culture system described in our previous study [28], and the medium was replaced every 2 days. At each predetermined time point, cell–gel constructs were sectioned into sheets matching with the chondral phase and the subchondral phase, respectively. The DNA content in these sheets was measured with a Quant-iT PicoGreen dsDNA kit (Invitrogen, Carlsbad, CA, USA) following the protocol provided by manufacturer [29].

### 2.11. Statistical Analysis

Data were expressed as mean ± standard deviation. Student’s *t*-test was used to compare the mean of independent groups. One-way ANOVA was employed to detect the difference between groups. Differences with statistical significance were determined when the *p*-value is less than 0.05.

## 3. Results

### 3.1. Characterization of NPs

**Figure 1:**
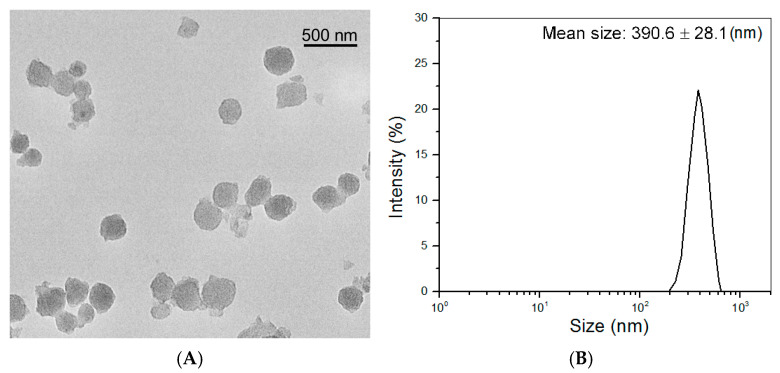
Representative TEM micrograph (**A**) and size distribution (**B**) of CH NPs.

CH NPs were prepared through a mild ionotropic gelation procedure using TPP as counterions, and so-produced CH NPs could have high ζ-potential [30], succinctly, more surface-exposed free amino groups. Thus, these surface amino groups of CH NPs can contribute to the network construction in composite gels via amino group-involved crosslinking reaction. Since these CH NPs will be used not only as a component for the preparation of composite gels but also as a potential carrier for loading hydrophilic drugs or bioactive molecules, both functions require limiting the swelling of CH NPs to some degree. Therefore, a small amount of PEGDE was used together with TPP to slightly crosslink these CH NPs. Figure 1 shows a representative TEM image for the achieved CH NPs. CH NPs showed a nearly spherical shape with good dispersion. They had a hydrodynamic mean size of about 390 nm and a ζ-potential of 43.7 (±2.08) mV, which are similar to that reported in the literature [23,29].

**Figure 2:**
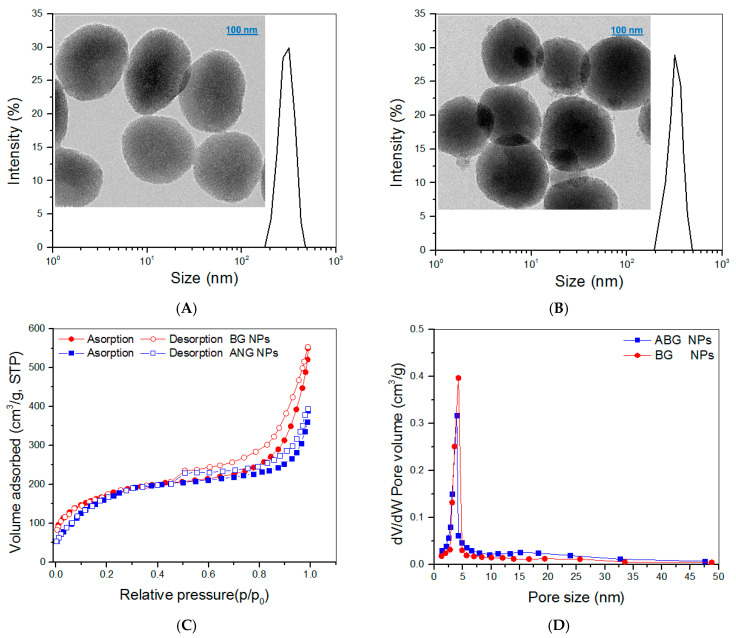
Size distributions and TEM images for BG NPs (**A**) and ABG NPs (**B**) and nitrogen adsorption–desorption curves (**C**) and pore-size distributions (**D**) for BG and ABG NPs.

The TEM image in Figure 2A displays that BG NPs had porous morphology and oviform or spherical shapes with well-defined dispersibility. These BG NPs had their hydrodynamic size distribution in an approximately symmetrical shape. The ABG NPs seemed to be more spherical than BG NPs (Figure 2B) and were also well-dispersive. The hydrodynamic size distribution of ABG NPs displayed a small difference in the peak value, and the distribution interval was shifted slightly towards the larger size, when compared to BG NPs.

The detected N_2_ adsorption–desorption isotherms for both BG and ABG NPs are presented in Figure 2C. Several characteristics can be drawn from these curves: the isotherms displayed typical hysteresis loops, the isotherms had a similar inception turning point of about 0.4 (p/p_0_), and the isotherm matching with BG NPs showed a significantly larger adsorbed volume in the higher pressure range in comparison with ABG NPs. These results suggest that both BG and ABG NPs have mesoporous pores inside, and, additionally, BG NPs have higher pore volume than that of ABG NPs [31,32]. Curves in Figure 2D exhibit that most of the pores in BG and ABG NPs had their size less than 8 nm and there was a small difference in the peak value between these two curves. Several sets of BG or ABG NPs were detected, and the relevant parameters are provided in Table 1. Data enumerated in Table 1 indicate the significant differences between ABG and BG NPs. Since BG NPs have a porous structure (Figure 2A), APTES can thus react with the hydroxyl groups that locate on the surface of BG NPs or reside on the surface of pores inside BG NPs [32,33]. As a result, ABG NPs have a significantly smaller surface area, pore volume, and pore size than those of BG NPs. BG NPs with hydroxyl group-exposed surfaces are known to show negative ζ-potential. The positive ζ-potential and larger hydrodynamic size for ABG NPs can be ascribed to their surface amino groups and APTES-resulted spacers, respectively.

### 3.2. Composite Hydrogels for Chondral Phase

**Figure 3:**
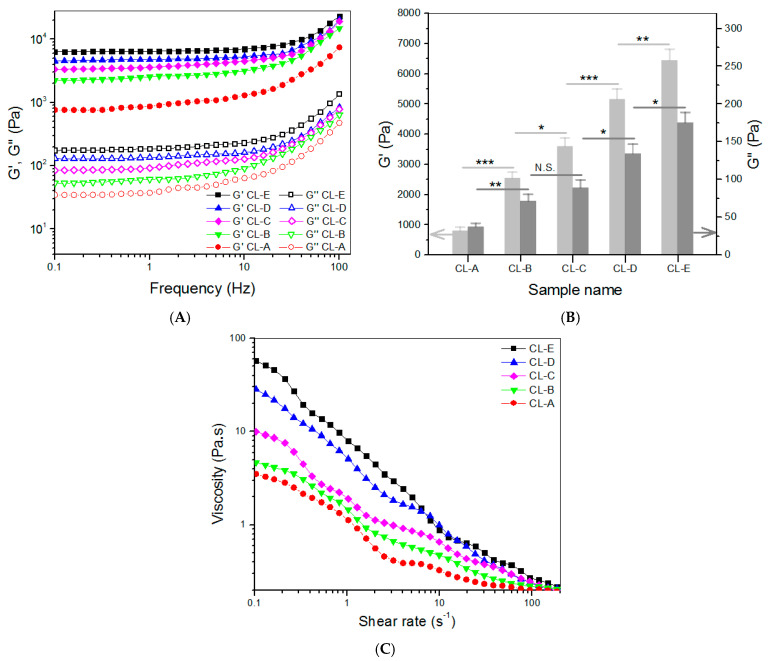
Frequency sweep spectra (**A**) of G′ and G″, average values (**B**) of G′ and G″ at 1 Hz, and viscosity versus shear rate ((**C**), 25 °C) for gels shown in Table 2 (* *p* < 0.05, ** *p* < 0.01, *** *p* < 0.001, N.S.—not significant).

CH, HA, and CH NPs were used to build hydrogels for intended use in the chondral phase of layered gel considering that HA is a typical component in the ECM of articular cartilage, and CH has its molecular structure similar to GAGs widely existing in the ECM. PEGDE was employed as a crosslinker for the preparation of gels, since its diepoxy groups have high reactivity towards amino and hydroxyl groups, and, accordingly, multiple networks can be built inside the resulting gels. Five kinds of composite gels were constructed, based on many preliminary experiments, and their parameters are summarized in Table 2. The employed amount of PEGDE was already optimized to a dosage of 0.1 (*w*/*v*)% for effectively crosslinking these gels and, meanwhile, enabling them to have sufficient safety. Frequency sweep spectra of G′ and G″ for these gels and the calculated average values of G′ and G″ at 1 Hz are presented in Figure 3.

In principle, the magnitude of G′ value in the linear viscoelastic region of gels can be used for assessing the strength of gels, and a strong gel usually has a large G′ value and a high G′/G″ ratio [34]. Figure 3A,B exhibit that the G′ value of these gels had a distinct upward trend, as the content of CH NPs increased. CL-C, CL-D, and CL-E gels had their G′ values of about 3.6, 5.2, and 6.5 kPa, respectively, being significantly higher than that of CL-B. These results indicate that the strength of CL-B gel can be remarkably enhanced by the addition of CH NPs. As mentioned earlier, CH NPs had a ζ-potential of around 43 mV, meaning that they contain many surface amino groups. The amino groups of CH NPs in CL-C, CL-D, and CL-E gels would contribute to the formation of multi-networks via PEGDE-bridged linkages, leading to their mechanical enhancement. Among these gels, CL-D and CL-E showed their G′ value of about 5.2 kPa or higher, and their G′/G″ ratio values were measured to be around 38.5 and 36.8, respectively, suggesting that CL-D and CL-E gels behave like mechanically strong gels [34,35]. Data in Table 2 indicate that there were significant differences in *ρ* values among these gels, and these gels changed their *ρ* values similarly to the variation trend of their corresponding G′. As shown in Table 2, these gels were crosslinked using the same amount of crosslinkers but differed from each other in compositions, and, significantly, in concentrations that are more succinctly correlated to the number of crosslinkable amino groups. Table 2 signifies that in the current formulations, the higher crosslinking degree for a gel can be ascribed to either an increase in the component or an increase in the content of CH NPs. Both increases contribute to more groups that can be crosslinked.

The gels shown in Table 2 were tested to examine their injectability, and curves for their viscosity versus shear rate are presented in Figure 3C. They were viscous in the low shear rate range and showed declining viscosity along with increasing shear rate. Their viscosity was lower than 1 Pa.s when the shear rate reached 10 s^−1^ or higher, indicative of their shear-thinning properties. Since gel precursor solutions are usually utilized at ambient temperature, Figure 3C signifies that these gels have well-defined injectability. Data in Table 2 and Figure 3B indicate that CL-E gel has a significantly shorter gelation time and a higher G′ value in comparison to CL-D, and hence, CL-E gel is selected for use in constructing the chondral phase of layered gel.

### 3.3. Composite Hydrogels for Subchondral Phase

**Figure 4:**
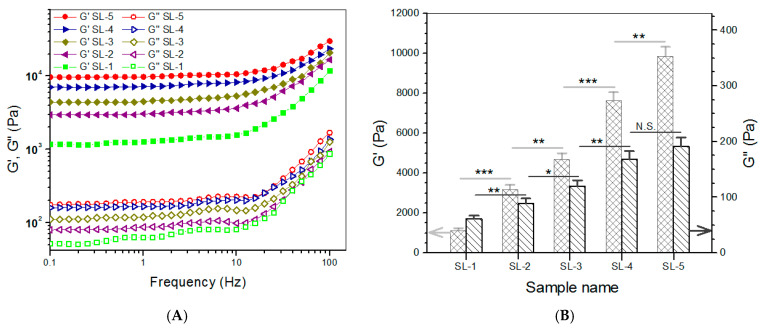

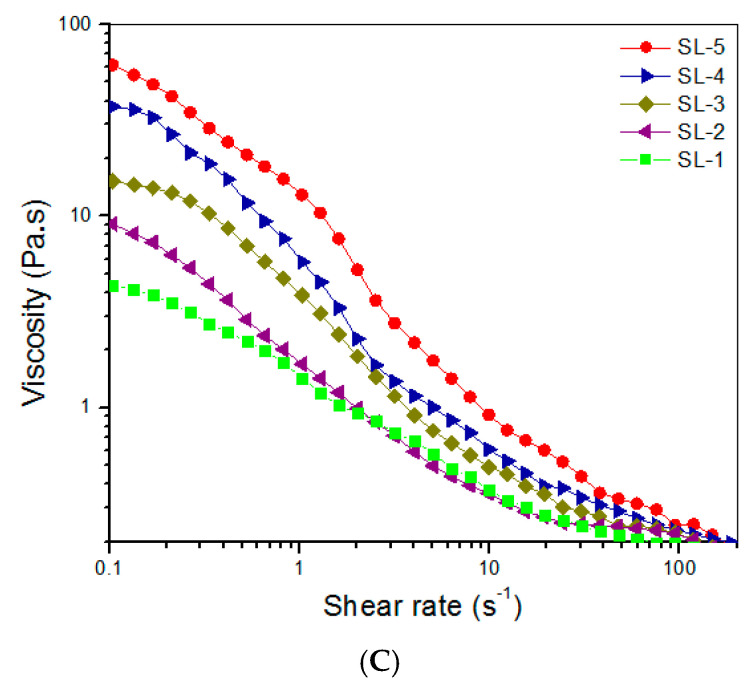
Frequency sweep spectra (**A**) of G′ and G″ and average values (**B**) of G′ and G″ at 1 Hz and viscosity versus shear rate ((**C**), 25 °C) for gels shown in Table 3 (* *p* < 0.05, ** *p* < 0.01, *** *p* < 0.001, N.S.—not significant).

CH, SF, and ABG NPs were employed to build hydrogels that are intended for use in the subchondral phase of layered gel. In these gels, the SF component in the gels was independently crosslinked using HRP and H_2_O_2_ as co-agents. Several kinds of composite gels were prepared with optimized formulations, and some parameters for them are provided in Table 3. Frequency sweep spectra of G′ and G″, average values of G′ and G″ at 1 Hz, and viscosity sweep spectra for these gels are presented in Figure 4. It can be observed that SL-2, SL-3, SL-4, and SL-5 gels had G′ values of about 3.1, 4.7, 7.6, and 9.9 kPa with matched G′/G″ ratio of 35.6, 39.4, 45.3, and 51.6, respectively. Like CH NPs, ABG NPs had free amino groups on their surface, indicated by positive ζ-potential (Table 1). These amino groups would thus be crosslinked by PEGDE and contribute to the construction of multi-networks. In comparison to CL-*i* (*i* = B, C, D, and E) gels, more networks can be built inside SL-*j* (*j* = 2, 3, 4, and 5) gels, because SF chains would build another network via HRP/H_2_O_2_ involved crosslinking. Consequently, SL-*j* (*j* = 2, 3, 4, and 5) gels showed markedly higher G′ values, when compared to their counterparts illustrated in Figure 3. As shown in Figure 4A,B, SL-3, SL-4, and SL-5 gels can be considered as mechanically strong gels due to their large G′ value and high G′/G″ ratio. It can be observed from Table 3 that the *ρ* values for these gels significantly changed with compositional variations. Similar to Table 2, the higher crosslinking degree for a gel in Table 3 can be attributed to either an increase in the component or an increase in the content of ABG NPs. In addition, it is noted that the gels in Table 3 had markedly higher crosslinking degrees than those in Table 2 when the gels in Table 1 and Table 2 are compared in a paired manner. These results are understandable, since the gels in Table 3 either have higher concentrations or contain additional crosslinkers. The curves in Figure 4C elucidate that these gels also showed shear-thinning characteristics, indicative of their injectability. Considering the significantly shorter gelation time and larger G′ value of SL-5 gel, compared to that of other gels, SL-5 gel is chosen for use in building the subchondral phase of layered gel.

### 3.4. Compressive Mechanical Property of Hydrogels

**Figure 5:**
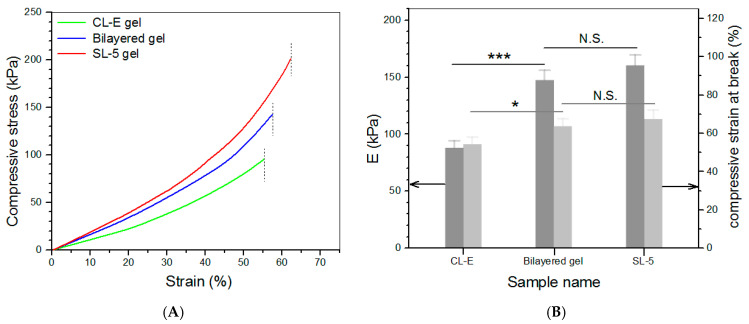
Strain–stress curves ((**A**), dash lines denote the compressive strain at the break) as well as average compressive modulus and strain at the break (**B**) (* *p* < 0.05, *** *p* < 0.001, N.S.—not significant).

**Figure 6:**
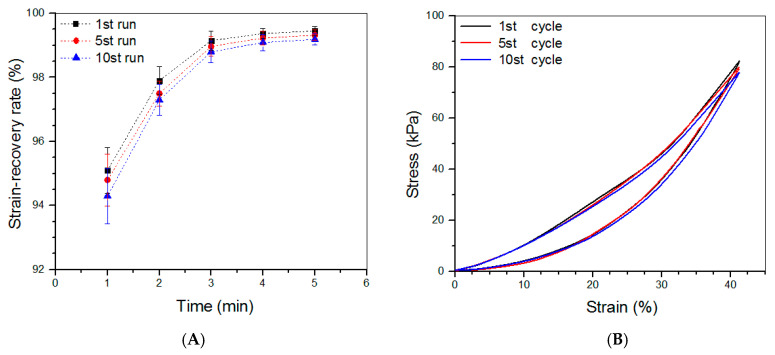
Strain-recovery rate (**A**) of bilayered gels that were compressed to 40% strain during loading–unloading tests and stress–strain curves (**B**) of bilayered gels during the 1st, 5th, and 10th compression runs.

Based on the above investigations, CL-E and SL-5 gels were used to build bilayered hydrogels, and some parameters for them are given in Table 4. To make comparisons among the top layer, bottom layer, and bilayered gels, some CL-E and SL-5 gel samples with a height of 4 mm and a diameter of 10 mm were also prepared for compression measurements. Three typical stress–strain curves for these gels are presented in Figure 5A. The stress of these gels showed upward trends with increasing strain, and their compressive strain at the break was higher than 50%. Several sets of samples for each kind of gel were measured to determine their average modulus and strain at the break, and the obtained data are depicted in Figure 5B. The E value of SL-5 gel reached about 160 kPa, and CL-E gel had an E value of around 85 kPa; these high E values again demonstrate that both SL-5 and CL-E gels are mechanically strong. It is worth noticing that the E value of SL-5 gel was almost twice that of CL-E gel, whereas the E value of bilayered gel was very close to that of SL-5 gel without significant difference, suggesting that the stronger layer governs the strength of the bilayered gel. Figure 5B also reveals that the bilayered gels had good elasticity, because their strain at the break is higher than 60%. The elasticity of bilayered gel was further examined by compressing them to 40% strain, and the detected strain recovery rate for them is graphed in Figure 6A. Data show that the height of bilayered gels quickly restored to about 94% within 1 min, and their height recovered around 99% in 5 min after compression 10 times, validating that these bilayered gels have high elasticity. Cyclic compression tests were conducted to investigate the toughness of bilayered gels, and the results are presented in Figure 6B. Curves in Figure 6B explicate that the stress continuously increased and reached about 80 kPa when the strain was extended to 40%, and a hysteresis loop with a complete return to the origin point was formed during the dimensional recovery in the first run. The following two hysteresis loops at 5th and 10th runs were seen to almost overlap with the hysteresis formed in the first cycle. The results shown in Figure 5 and Figure 6 demonstrate the bilayered gels are mechanically strong, elastic, and tough.

### 3.5. Porous Parameters of Dry Bilayered Gels

**Figure 7:**
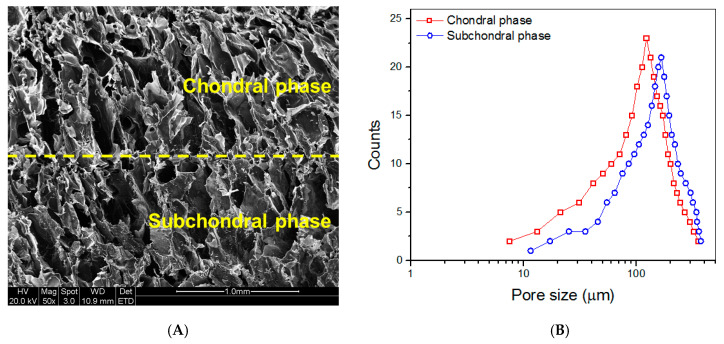
SEM image taken from the vertical section of dry layered gel ((**A**), yellow dashed line in the image indicates interface between two phases) and pore-size distributions for chondral and subchondral phases (**B**).

Figure 7A presents a representative SEM image taken from the vertical section of the dry layered gel. Both chondral and subchondral phases were observed to be highly porous with well-interconnected porous architecture. Two phases were measured to have wide pore-size distributions (Figure 7B). Data for the calculated mean pore size and average porosity of chondral and subchondral phases are listed in Table 4. Figure 7B and Table 4 indicate that there were non-significant differences in the pore-size distributions, mean pore-size and average porosity between chondral and subchondral phases.

### 3.6. Release Profiles of Ions

**Figure 8:**
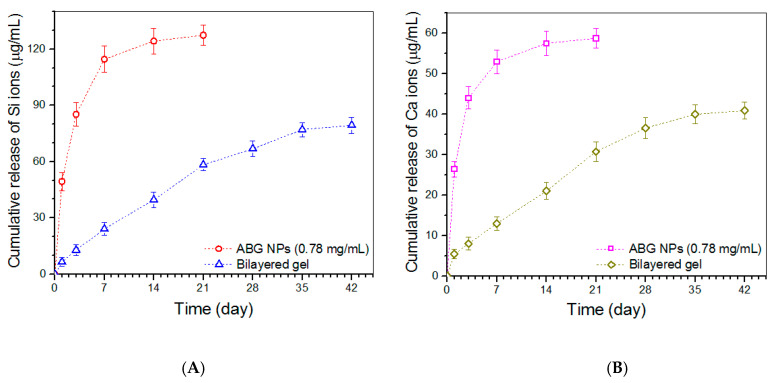
Release profiles of Si (**A**) and Ca (**B**) ions for ABG NPs and bilayered gel samples.

Release profiles of Si ions for ABG NPs and bilayered gel are presented in Figure 8A. ABG NPs showed a marked burst release feature, and their cumulative release reached a level higher than 110 μg/mL in the first week. In contrast to ABG NPs, the bilayered gel behaved in a quite different way. The gel released about 6 μg/mL on the first day, and afterward, their release curve exhibited an approximately linear upward trend for around three weeks. A similar burst release of Ca ions from ABG NPs was also detected, and the bilayered gel showed the capacity to administer the Ca-ion release in an approximately linear manner for about four weeks (Figure 8B).

### 3.7. Cell Growth

**Figure 9:**
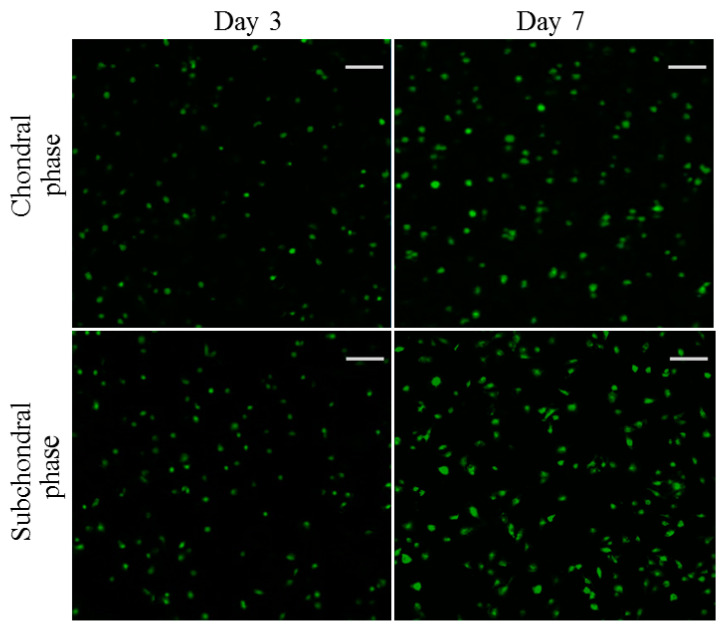
Confocal micrographs for stained chondrocytes seeded in chondral phase of the layered gel and osteoblasts seeded subchondral phase of the layered gel for various durations (green: viable cells; red: dead cells; scale bar: 100 μm).

Chondrocytes and osteoblasts were seeded in the chondral and subchondral phases of the bilayered gel, respectively, and cultured for various durations of up to 7 days to evaluate their viability. Figure 9 displays representative fluorescence micrographs for the stained chondrocytes and osteoblasts. Very few dead cells were detected from both chondral and subchondral phases of the bilayered gel after a 3- or 7-day culture. In addition, in both phases of the bilayered gel, the cell density in the images matching with the 7-day culture was significantly higher than that corresponding to the 3-day culture. These images reveal that the seeded chondrocytes and osteoblasts were highly viable, and both phases had good biocompatability although there are differences in composition and cross-linker.

**Figure 10:**
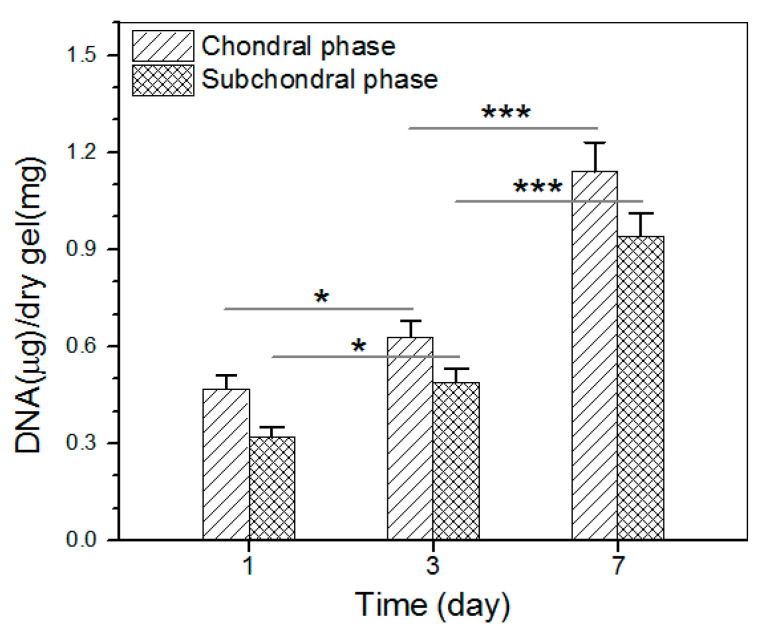
Proliferation of chondrocytes and osteoblasts that were seeded in chondral phase and subchondral phase of the layered gel, respectively (* *p* < 0.05, *** *p* < 0.001).

The proliferation of chondrocytes and osteoblasts that were seeded in the chondral and subchondral phases of the bilayered gel was measured, and results are explicated in Figure 10. The bar graph indicates that the growth of chondrocytes and osteoblasts roughly experienced two stages: fewer cells grew from day 1 to day 3, and cells grew fast from day 3 to day 7. The slow cell growth in the first stage can be ascribed to the cell population recovery, and the second stage indicates the occurrence of cell proliferation in terms of increasing DNA amount.

## 4. Discussion

Articular cartilage is an elastic and tough tissue that overlies the articulating bony ends in diarthrodial joints. It fulfills critical biomechanical functions for increasing joint congruence, protecting the subchondral bone from high stresses and reducing friction at the edge of long bones [36]. The response of many tissues to injury usually follows necrosis, inflammation, repair, and remodeling cascade of events, but cartilage repair does not undergo these processes [2]. Articular cartilage has a stratiform structure, and it can be distinguished into four diacritical layers: superficial, middle, deep, and calcified cartilage layers [15]. Cartilage defects can occur in the superficial layer or extend to the deep layer, and even affect the underlying subchondral bone. Some articular cartilage defects do not start directly from the chondral phase, and they are secondarily caused by subchondral bone disease or injury [2]. It is very difficult to effectively repair a defect located only in the chondral phase of articular cartilage, because this tissue lacks access to a pool of potential reparative cells and growth factors [2,3], and thus, cartilage lesions are generally irretrievable [2,3,36,37,38]. At present, there has been increasing awareness that the repair and regeneration of articular cartilage should be performed by considering hyaline cartilage and subchondral bone as integrated units [2,3,37,38,39,40].

Since the articular cartilage is about 4 mm thick or less, attempts to simulate the ECM of the articular cartilage by using scaffolds with precise structure and composition are extremely difficult [1,3,14,15]. The bilayered scaffolds with varying compositions have thus been explored for use in the repair and regeneration of osteochondral tissues, since they can roughly mimic the structure and properties of ECM in the osteochondral unit [4,39,40,41]. Many studies have been performed on solid-state bilayered scaffolds so far [1,2,3,4,14,15,37,38,39,40,41]. Despite their applicability, the utilization of solid-state bilayered scaffolds for osteochondral repair faces several difficulties. They are usually unsuitable for use in the repair of osteochondral defects with complex shapes, and the complex defect generally needs to be trimmed into the circular hole to fit the cylindrical scaffold. Such pre-treatment would result in enlarged defect size and often cause damage to the surrounding tissue. Even so, the cylindrical scaffold that is implanted into the osteochondral defect also has difficulty in forming seamless connections with the host tissue. Moreover, the implanted scaffold has to be fixed by suturing with cartilage or perichondrium or other techniques.

In the search for alternative materials for the repair and regeneration of osteochondral tissues, bilayered hydrogels with or without cell-loading have gained increasing interest in this field [6,7,41,42,43,44,45]. The injective hydrogels can fill the osteochondral defects with arbitrary morphologies while forming a bilayered structure in situ without additional pre-treatments. Since a bilayered hydrogel for use in osteochondral repair practically acts as a 3-dimensional temporary ECM-like support for housing different cells and facilitating their growth, proliferation and matrix synthesis, the property of bilayered hydrogel can thus impose substantial impacts on the structure and function of neonatal osteochondral tissues. It is now realized that the bilayered hydrogels intended for osteochondral repair should be strong, elastic, and tough while promoting the growth of cells, because the seeded or recruited cells actively respond to the mechanical performance of the gels during their growth and ECM synthesis [43,44,46]. In this study, the multi-network strategy has been employed for building a new type of bilayered hydrogel. In both chondral and subchondral phases of the bilayered hydrogel, GP was used to bring the pH of gels back to neutrality while functioning as a physical crosslinker; PEGDE was utilized as a chemical crosslinker to crosslink amino or hydroxyl groups of components, since diepoxy groups in PEGDE are highly reactive towards amino or hydroxyl groups [47,48]. Thus, multi-networks can be established inside the chondral phase of bilayered gel via PEGDE-bridged linkages. In the subchondral phase of bilayered gel, besides the PEGDE-bridged multi-networks, an additional network was built by the SF chains via tyrosine residue crosslinking. As a result, the achieved bilayered gels have gained strong, elastic, and tough properties, as indicated in Figure 5 and Figure 6.

Considering that GP, PEGDE, and HRP/H_2_O_2_ were used for the construction of bilayered gels, these gels were tested to identify if they are safe and suitable for supporting the growth of seeded cells. The results in Figure 9 and Figure 10 verify that bilayered gels are competent for the growth of seeded chondrocytes and osteoblasts. As far as CH NPs and ABG NPs, in addition to participating in the construction of multi-networks through their surface amino groups, they also impart the bilayered gels with the potential for loading certain drugs or active molecules. Although CH NPs used in this study were prepared through an ionotropic gelation method using TPP, they were also slightly crosslinked by PEGDE. So-produced CH NPs have a limited swelling degree, allowing them to effectively enhance the composite gels while serving as a carrier. ABG NPs are porous with high porosity (Table 1), and they can also act as a potential vehicle. In addition, ABG NPs themselves can release Si and Ca ions, and hence, ABG NPs incorporated in the subchondral phase of bilayered gel would assist in the integration of neonatal bone tissue with host tissue [47].

So far several studies have been conducted to develop bilayered hydrogels for osteochondral repair applications by using alginate, oligo(poly(ethylene glycol) fumarate), gellan gum, polyvinyl alcohol, collagen, glutamic acid, bacterial cellulose, SF, CH, and gelatin alone or in their combination [41,42,43,44,45,49,50,51,52,53]. Very few of these studies have jointly investigated the strength, elasticity, and toughness of their bilayered hydrogels, and none of them has employed BG NPs or ABG NPs as a component in the subchondral layer of bilayered hydrogels. The bilayered gel presented in the present study differs from those bilayered hydrogels reported in the literature in the strong, elastic, and tough characteristics as well as the ability to control the release of bioactive ions.

Based on the above investigations, it can draw that these bilayered gels would be conducive to the repair and reconstruction of osteochondral defects in terms of their structure, composition, and property. The present study was conducted to examine the mechanical properties and biocompatibility of the bilayered gels. More studies on their in vivo applications in osteochondral repair and regeneration are underway. The relevant results will be summarized in other reports.

## 5. Conclusions

A new type of bilayered composite gel was successfully constructed using CH, HA, SF, CH NPs, and ABG NPs as components while employing GP as a physical crosslinker, PEGDE as a chemical crosslinker, and HRP/H_2_O_2_ as crosslinking co-agents. The surface amino groups of CH NPs and ABG NPs significantly contributed to the formation of multi-networks in the chondral and subchondral phases of bilayered gels. The bilayered gels with optimally formulated compositions were found to be mechanically strong, elastic, and tough. Moreover, these bilayered gels exhibited the ability to release Si or Ca ions in approximately linear manners for a few weeks. They were demonstrated to be injectable and capable of supporting the in-growth and proliferation of chondrocytes and osteoblasts that were seeded in the chondral and subchondral phases of gels, respectively. The results reveal that these bilayered gels have the potential for osteochondral repair applications.

## Figures and Tables

**Table 1:** Parameters for two types of nanoparticles.

Sample Name	Surface Area (m^2^/g)	Pore Volume (mL/g)	Pore Size (nm)	ζ-Potential (mV)	Particle Size (nm) ^(b)^	Content of Amino Groups (mmoL/g)
BG ^(a)^	738.6 ± 37.2	1.39 ± 0.08	4.37 ± 0.13	−12.7 ± 0.93	303.8 ± 21.6	-
ABG	541.2 ± 21.9	0.92 ± 0.06	3.85 ± 0.11	29.4 ± 1.42	364.1 ± 19.2	0.496 ± 0.029

^(a)^ CaO/SiO_2_ molar ratio for BG NPs was detected to be around 0.14 from their energy dispersive spectra. ^(b)^ Hydrodynamic size was detected via DLS measurements.

**Table 2:** Parameters for composite hydrogels composed of CH, HA, and CH NPs ^(a,b)^.

Sample Name	CH (*w*/*v*%)	HA (*w*/*v*%)	CH NPs (*w*/*v*%)	PEGDE (*w*/*v*%)	Gelation Time (min) ^(c)^	Φ	*ρ* (×10^−6^ mol/cm^3^) ^(d)^
CL-A	1.8	-	-	0.1	9.25 ± 19	0.0185	1.2263
CL-B	1.8	0.6	-	0.1	16.75 ± 0.95	0.0181	3.9191 *
CL-C	1.8	0.6	0.5	0.1	12 ± 0.81	0.0235	5.0746 ^#^
CL-D	1.8	0.6	1.0	0.1	7.5 ± 0.57	0.0275	6.8978 ^†^
CL-E	1.8	0.6	1.5	0.1	4.25 ± 0.5	0.0337	8.0597 ^✞^

^(a)^ The feed amount of each component in the gel was calculated by 1 mL of gel volume. ^(b)^ The concentration of GP for gel samples was 5.6 (*w*/*v* %). ^(c)^ Gelation time was estimated by inverting the vials every 1 min. ^(d)^ * *p* < 0.001, compared to CL-A; ^#^ *p* < 0.05, compared to CL-B; ^†^ *p* < 0.05, compared to CL-C; ^✞^ *p* < 0.05, compared to CL-D.

**Table 3:** Parameters for composite hydrogels composed of CH, SF, and ABG NPs ^(a,b)^.

Sample Name	CH (*w*/*v*%)	SF (*w*/*v*%)	ABG NPs (*w*/*v*%)	PEGDE (*w*/*v*%)	H_2_O_2_ (μL)	HRP (μL)	Gelation Time (sec) ^(c)^	Φ	*ρ* (×10^−6^ mol/cm^3^) ^(d)^
SL-1	2.0	-	-	0.1	-	-	525 ± 17	0.0199	1.8245
SL-2	2.0	1.0	-	0.1	10	10	450 ± 24	0.0265	4.2963 *
SL-3	2.0	1.0	0.5	0.1	10	10	330 ± 24	0.0264	6.3582 ^#^
SL-4	2.0	1.0	1.0	0.1	10	10	255 ± 17	0.0265	10.3221 ^†^
SL-5	2.0	1.0	1.5	0.1	10	10	195 ± 17	0.0268	13.2961 ^✞^

^(a)^ The feed amount of each component in the gel was calculated by 1 mL of gel volume. ^(b)^ The concentration of GP for gel samples was 5.6 (*w*/*v* %). ^(c)^ Gelation time was estimated by inverting the vials every 30 s. ^(d)^ * *p* < 0.001, compared to SL-1; ^#^ *p* < 0.05, compared to SL-2; ^†^ *p* < 0.001, compared to SL-3; ^✞^ *p* < 0.01, compared to SL-4.

**Table 4:** Parameters for bilayered hydrogel.

Layer Name	Component ^(a)^	Thickness (mm)	Diameter (mm)	Porosity (%)	Pore Size (μm)
Top layer (chondral phase)	CL-E	2	10	70.1 ± 3.1	135.2 ± 11.7
Bottom layer (subchondral phase)	SL-5	2	10	75.3 ± 4.2	150.3 ± 13.1

^(a)^ See Table 2 and Table 3 for parameters of these two components.

## Data Availability

The data presented are available on request from the corresponding author.
